# Evaluation of the challenges faced in increasing contraceptive access within a community college population

**DOI:** 10.1186/s40834-017-0051-8

**Published:** 2017-10-23

**Authors:** Jacqueline Lamme, Alison Edelman, Emily Padua, Jeffrey T. Jensen

**Affiliations:** Department of Obstetrics & Gynecology, Oregon Health & Science University, 3181 SW Sam Jackson Park Road, UHN 50, Portland, OR 97239 USA

**Keywords:** Contraception, LARC, Access, Community college

## Abstract

**Background:**

Research demonstrates removing barriers to access, decreasing costs and offering same-day placement of long-acting reversible contraception (LARC) increases contraceptive uptake in young women. For those in community college (CC), LARC utilization might reduce the risk of dropout and improve degree completion. We identified a local school who had documented an unmet need for on-campus services through a recent student assessment. We then established an on-campus, same day contraceptive clinic at the CC as part of a clinical trial. We found that students did not use the service even after multiple attempts to increase awareness and we ended the study. Here, we report lessons learned from attempting research in this environment in addition to results from a follow-up survey to determine why students did not access the clinical resource. Students reported that they already had good access to contraception and preferred to get their healthcare off-campus. This study demonstrates the complexities of studying highly focused interventions to influence access to care in the current health care environment with ever changing regulations.

**Trial registration:**

NCT02735551 **.** Registered April 6, 2016.

Approximately half of the pregnancies in the United States are unintended with almost half of unintended pregnancies ending in abortion [1, 2]. Long acting reversible contraceptive (LARC) methods are known to be more effective than shorter acting methods and by increasing their use in a population, the abortion rate falls [3]. Although studies have demonstrated an increase LARC uptake when barriers to access and cost were removed [1], their use is less likely in women of lower socio-economic status [4].

The community college (CC) population is diverse and students tend to be within a lower socioeconomic bracket compared to four-year college students [5]. National data from 2016 shows that 31% of Community College students come from families in the lowest quintile family-income bracket, a disproportionate representation [6]. U.S. Department of Education statistics indicate that nearly 50% of community college students drop out within 6 years of enrolling [7]. For those in community college, LARC might aid in increased school retention and completion secondary to a better ability to plan and prevent pregnancy. Simply removing cost barriers may not be enough to increase LARC usage among high-risk populations. Additional barriers women might face include taking time off work, finding child-care if needed and transportation logistics and cost. Bednarek et al. showed that 30% of women, who expressed interest in a LARC method and did not obtain the method same day during a family planning visit, do not return to the clinic for IUD placement [8]. A 2016 survey by the American College Health Association showed that only 55% of college students at risk for pregnancy utilized contraception and this primarily consisted of less effective methods [9]. Additionally, most research at this time focuses on contraceptive use within traditional 4-year colleges and is not specific to community college students. Prior research has noted that a lack of contraceptive knowledge may lead to incorrect use or nonuse of effective birth control methods in college students [10]. However, while four-year colleges have on campus student health clinics (SHCs), many community colleges do not, which may make it even more difficult for students to obtain contraceptive care and initiate a LARC method without a referral off campus. Emerging research among community college students reveals a lack of accurate knowledge surrounding contraception which may further limit usage [11].

We hypothesized that the availability of on-campus contraceptive counseling and services with same day LARC placement would increase the proportion of students using a LARC method. By introducing these services, we anticipated a decrease in unplanned/mistimed pregnancies, which could lead to an increased 2-year certificate completion rate.

## Clinic intervention study

We reached out to multiple community colleges in Oregon during the development stages of the study and chose to collaborate with Mount Hood Community College (MHCC) in Gresham, Oregon as they demonstrated a high interest at both the student and administrative level to establishing services on campus. MHCC is located at the urban-rural divide and the majority of their students have an annual income of less than $25,000. Twice as many male students as female students obtained their 2-year certificate in 2014. The student government had demonstrated need for health care through a 2015 survey of the student body. Approximately 1000 students had participated in that online questionnaire. Questions included if students had health insurance, household income and what types of healthcare services they felt were important. They also asked “If MHCC were to partner with a medical provider that offers healthcare services to students and their families at substantially reduced prices, how likely would you be to use the service?” They found that a majority (78%) of students surveyed wanted healthcare on campus. Reproductive health services were identified as either important or very important by the students to be included in the health services. Additionally, MHCC had a well utilized Planned Parenthood (PP) Satellite Clinic located on campus in the past, which was closed when PP clinical services were consolidated to a few primary sites. Finally, at the time of planning our intervention, the MHCC Student Government was evaluating different avenues that would allow them to re-introduce clinical services for students on campus.

After obtaining approval from the OHSU IRB, securing funding for the study, and negotiating donation of no-cost LARC devices for study participants, we established an on-campus contraceptive counseling and services clinic in September 2016 with the support of the MHCC administration. All participants watched a standardized, tier-based contraceptive counseling video (http://larcfirst.com/sessions.html). Our research intervention was the randomization of students who desired a LARC method after receiving contraceptive counseling to either same day placement or referral to a nearby community clinic. Those desiring short-acting contraceptive methods would receive a same day prescription. We planned to assess a variety of secondary outcomes (including satisfaction, continued usage, unintended pregnancies and student status) over a 12-month period to determine whether having an on-site clinic with same day provision of contraceptive services at this location improved a variety of outcomes for students. We expected that following participants longitudinally would allow us to assess additional secondary educational outcomes. Limitations of our approach included the short follow up time and the lack of an existing health clinic on campus serving the student body at the time of the study. Although we successfully created a clinic space on campus, due to space constraints, the location provided by the college was not central, decreasing visibility of the space.

After 3 months of recruiting, including flyers, newspaper articles, advertisements, tabling events, listserv emails, and participation in student government meetings, only 3 participants enrolled and recruitment was closed. All 3 participants chose short acting birth control (SARC) methods. We were forced to close enrollment for the study.

## Evaluation study

After closing enrollment for our primary study, we developed a brief questionnaire to provide descriptive data on possible reasons why the on-campus clinic was not utilized as expected. After receiving OHSU IRB and MHCC administration approval, we distributed a link to an electronic survey to all students. The Qualtrics platform was utilized to maintain the confidentiality of respondents. Only full-time students were eligible to have their data included in the analysis and the survey link remained open for 30 days. Questions included brief demographic data as well as questions on how they learned about activities on campus, if they had heard of the on-campus clinic, if they were currently using birth control, what type of birth control they used and if they would prefer to get their healthcare on campus.

We recruited a convenience sample of 153 students who accessed the link in the 30-day window. Of these, 112 were currently enrolled in a certificate or degree program and eligible to participate. Most of the students who answered the question were female and between the ages of 17–30. The majority (91/110, 82%) had health insurance. Students reported learning about campus activities primarily from e-mail (85%), flyers (49%) and word of mouth (30%). Only 15/110 (13.64%) students were aware of the research clinic even though we had used all of the methods of recruiting cited by the students. Six of these 15 students were already using a birth control method. Of all student responders, 53/110 (48.18%) were using a contraceptive (45% pills, 26% IUD, 11% Implant), mostly obtained from their primary care provider (*n* = 43/94, 46%) or Planned Parenthood (*n* = 13/94, 13.83%). The majority of respondents (*n* = 66/105, 63%) stated that they preferred not to get their healthcare on campus.

## Lessons learned

Although this CC had demonstrated student need and desire for on-campus reproductive health services, there was limited utilization of an on-campus clinic. In our follow-up survey, we discovered that most students at this campus reported good access to contraception from off-campus providers and that they actually prefer not to receive reproductive healthcare on campus. Anecdotally, during tabling activities to aid recruitment, many students stopped by to express their support for contraceptive services but in the same breath also reported that they already had birth control they were happy with. A few students reported they were not interested in enrolling because they were worried that the study was utilizing experimental methods of contraception. This misunderstanding may have occurred because of the wording of recruitment flyers or misperceptions from the articles published in the student newspaper. If this study was attempted at a location with an on-campus health clinic utilized by the students, it is possible that improved recruitment would be noted.

Possible explanations include changes to health care funding that occurred in the two years that passed from the MHCC needs assessment. Both the Affordable Care Act (ACA) and Oregon’s Contraceptive Care (CCare) program increased federal and local health care support to low-income students, and may have greatly changed the landscape of the need for services on campus. Economic models estimated that the expansion of contraceptive services in Oregon with the initiation of the ACA would increase coverage of contraceptive services anywhere from 12%–25% [12]. In Oregon, women who do not have other insurance coverage for contraception and make less than $566/week are eligible for coverage for any method of contraception through a program referred to as Oregon Contraceptive Care (CCare) [13]. A recent retrospective cohort of 344, 856 adolescents accessing CCare clinics in Oregon demonstrated that adolescents were as likely as older women to transition from no method or less effective methods of contraceptive to more effective methods when accessing this care [14]. Additionally, during the time period of our intervention, the enhanced contraception coverage available through the ACA was widely discussed and advertised through both social and mainstream media. Furthermore, the initial MHCC needs assessment did not specifically ask students if they already had off-campus access to services. Future similar efforts would likely benefit from a directed needs assessment specific to the study intervention rather than relying purely on an internal questionnaire.

## Conclusion

The lack of clinic utilization was likely a complex combination of increased access to contraceptive services in Oregon, increasing awareness of highly effective contraceptive methods among young adults and increasing overall LARC utilization within Oregon. These various aspects of this study and the challenges is faced demonstrate that studying targeted interventions to influence access to care in the current health care environment with its ever-changing regulations, is challenging.
